# The bayberry database: a multiomic database for *Myrica rubra*, an important fruit tree with medicinal value

**DOI:** 10.1186/s12870-021-03232-x

**Published:** 2021-10-06

**Authors:** Haiying Ren, Yuanhao He, Xingjiang Qi, Xiliang Zheng, Shuwen Zhang, Zheping Yu, Fengrong Hu

**Affiliations:** 1 Institute of Horticulture, Zhejiang Academy of Agricultural Sciences, Hangzhou, 310021 China; 2 College of Landscape Architecture, Nanjing Forestry University, Nanjing, 210037 China

**Keywords:** Chinese bayberry, Database, Genome, Transcriptome, Tools, Germplasm resource

## Abstract

**Background:**

Chinese bayberry (*Myrica rubra* Sieb. & Zucc.) is an important fruit tree in China, and has high medicinal value. At present, the genome, transcriptome and germplasm resources of bayberry have been reported. In order to make more convenient use of these data, the Bayberry Database was established.

**Results:**

The Bayberry Database is a comprehensive and intuitive data platform for examining the diverse annotated genome and germplasm resources of this species. This database contains nine central functional domains to interact with multiomic data: home, genome, germplasm, markers, tools, map, expression, reference, and contact. All domains provide pathways to a variety of data types composed of a reference genome sequence, transcriptomic data, gene patterns, phenotypic data, fruit images of *Myrica rubra* varieties, gSSR data, gene maps with annotation and evolutionary analyses. The tools module includes BLAST search, keyword search, sequence fetch and enrichment analysis functions.

**Conclusions:**

The web address of the database is as follows http://www.bayberrybase.cn/. The *Myrica rubra* database is an intelligent, interactive, and user-friendly system that enables researchers, breeders and horticultural personnel to browse, search and retrieve relevant and useful information and thus facilitate genomic research and breeding efforts concerning *Myrica rubra*. This database will be of great help to bayberry research and breeding in the future.

## Background

The Myricaceae family consists of three genera, including approximately 50 species, and it is mainly distributed in warm and humid areas of Asia (e.g., India, Japan, and China), South America (e.g., Brazil), North America (e.g., the USA), Africa (e.g., Kenya), and Europe (e.g., Spain, Switzerland, Norway, and France) [1–4]. Chinese bayberry (*Myrica rubra* Sieb. & Zucc.) is a fruit tree with high economic value, with a cultivation area in China of approximately 334,000 ha and an annual yield of approximately 950,000 t. In addition, many kinds of organs of Chinese bayberry are applied in traditional Chinese medicine. Chinese bayberry extracts contain antioxidants that may serve to alleviate health issues such as diarrhea, inflammation, and cancer [1, 5].

With rapid advances in genome technology, obtaining high-quality genome assemblies of most plant species is no longer difficult [6, 7]. Through genome sequencing, a 289.92 Mb genome for fine mapping was assembled with 26,325 predicted protein-coding genes [8, 9]. To elucidate the evolution and antioxidant activity of Chinese bayberry, we performed in-depth transcriptomic analysis to determine the genes that regulate antioxidant and pharmacological activities [8, 9]. The paper provides some vital data for the genetic improvement of bayberry in the future and is conducive to the understanding of its genetic evolution.

Fruit quality is also a significant agricultural trait of bayberry and is difficult to dissect. Fruits with a distinctive flavor and color are more popular on the market. The color of bayberry fruit is primarily determined by the level of anthocyanin accumulation, which exists stably in plant cells, making the fruit purple or red [10, 11]. In addition to fruit color, total sugar content is the primary index of fruit flavor quality [12]. Sugar affects the flavor of the fruit and is the primary source of energy. Mature bayberry fruit primarily contains sucrose, fructose and glucose; of these sugars, sucrose accounts for the highest proportion [13]. The key period of color development of red bayberry is the whiting stage, after which the sugar components accumulate rapidly, and the total sugar content reaches the maximum at the harvest stage, when the fruit flavor quality is the best [12, 14]. Moreover, research findings suggest that xenia affects fruit yield and quality, and various pollen types have a marked impact on the quality of bayberry fruit [15, 16]. Research on this phenomenon has practical and immediate significance for the fruit tree industry.

With the rapid development of genomic technology and bioinformatic data, determining how multiomics data should be stored, interlinked and presented in one interface requires a sustained effort. From a researcher’s point of view, some user-friendly tools, such as genome browsers, cultivar banks, genetic maps and design tools to assist selection, are necessary. Currently, there are several websites for plant research that are publicly available, such as the Cucurbitaceae database (GourdBase, http://www.gourdbase.cn/) [17] and Malvaceae database (MaGenDB, http://magen.whu.edu.cn) [18], but there is no publicly available database for bayberry. Collecting essential molecular breeding data, such as genome, transcriptome, genetic mapping and phenotypic information, of bayberry remains difficult. Therefore, it is urgently important to establish an integrated, professional and intelligent bayberry database to enable breeders, researchers and horticulturalists to retrieve and access many kinds of necessary data.

In this paper, a special database named the Bayberry Database fills the gap in the body of knowledge for significant plant genera, integrates large-scale multiomics data, implements integrative data visualization methods, and establishes a new functional comparison system to facilitate the research on molecular breeding and biological traits of bayberry. The primary purpose of this multifunctional database is to provide a reference genome resource, as well as germplasm (cultivars, fruit shape, and disease resistance), molecular marker and transcriptomic data, to enable marker-assisted breeding of bayberry. In addition, the Bayberry Database also features hundreds of fruit shape images, which is of considerable value to the study of bayberry fruit shape.

## Utility and discussion

### Bayberry database content

**Fig. 1:**
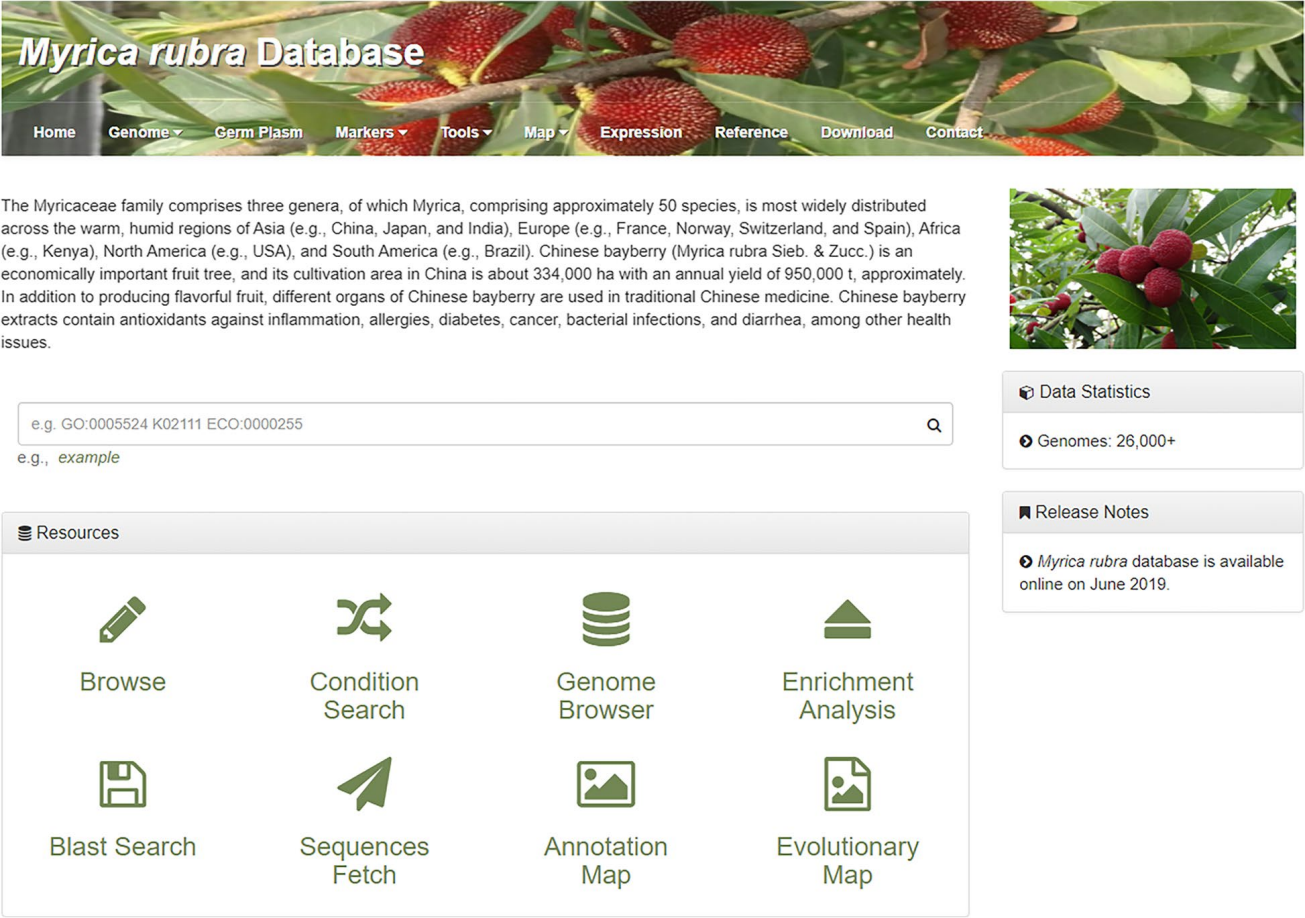
Bayberry Database homepage. Nine main modules are displayed at the top of the interface and include the following: the genome, germplasm (phenome), markers, tools, maps, expression, reference, download and contact modules

The Bayberry Database contains nine central functional domains: home, genome, germplasm, markers, tools, map, expression, reference, and contact (Fig. 1). At present, the Bayberry Database contains the following information: 26,325 genes with tissue expression patterns, fruit shape phenotypes for 102 diverse accessions, basic characteristics of 59 gSSR markers, transcriptomic data of 13,706 genes, and 2 genome maps of bayberry. The Bayberry Database also features a reference module in which 137 papers providing the above information can be freely browsed and downloaded with the authors’ permission. Based on the above information, this database and its related tools will enable users to quickly retrieve large-scale functional information for biological research.

### Application of the bayberry database

#### Genome search

**Fig. 2:**
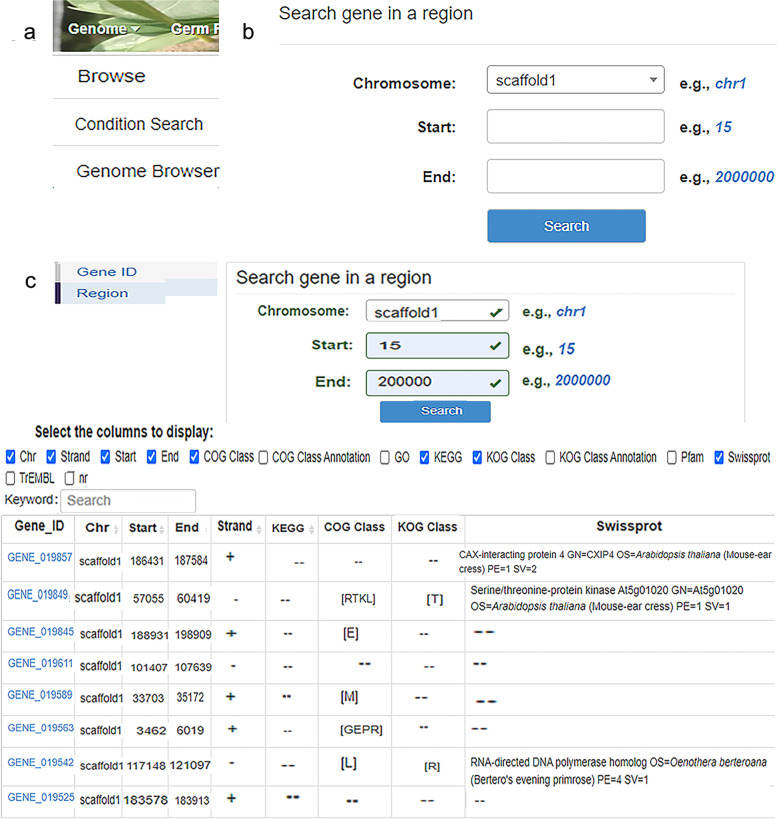
Detailed functional view of the genome module. **A** ‘Genome’ includes ‘Browse’, ‘Condition Search’, and ‘Genome Browser’. **B** Demonstration of the ‘Search By Region’ dialog box. **C** An example of the search result

The bayberry genome database offers the whole genome information of bayberry recently compiled by the authors’ laboratory. The size of the whole genome of bayberry is approximately 289.92 Mb [8], which is considerably smaller than the genomes of apple (742 Mb) [19] and pear (527 Mb) [20]. When the user clicks on the ‘Genome’ heading, the column header label displays the ‘Gene Browser’, ‘Condition Search’, and ‘Browse’ subheadings (Fig. 2A). The user can access the sublinks and retrieve the required data by clicking on any one of these labels. For example, if the user clicks on ‘Conditional Search’, an additional layer will appear with two options that read ‘Gene ID’ and ‘Region’. After clicking on the ‘Region’ option, ‘Chromosome’, ‘Start’ and ‘End’ are displayed (Fig. 2B). After entering the query information in the box, the user can then click on the ‘Search’ button, and a large quantity of data will display quickly according to the requirements (Fig. 2C).

#### Genome browser

**Fig. 3:**
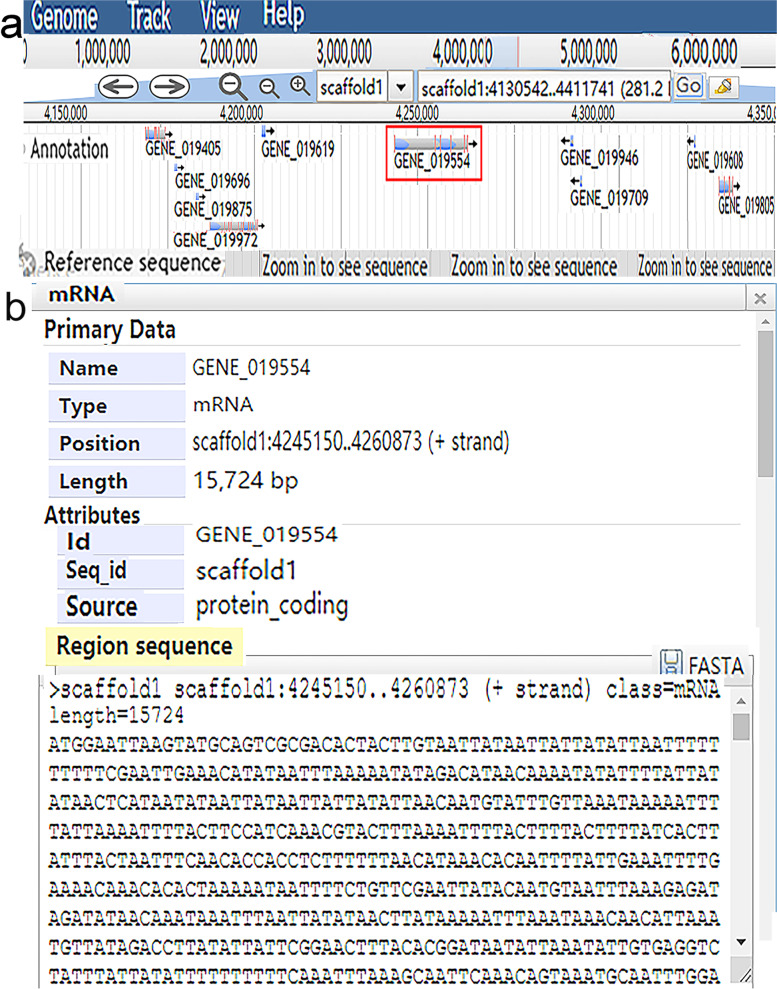
Regional view of the genome using the Bayberry Database genome browser. **A** A graphic view of the region 4,130,542 bp to 4,411,741 bp on Scaffold1. **B** The interface after clicking on ‘GENE_019554’

The genome browser of the Bayberry Database comprises tracks describing the gene sequence, gene structure, mRNAs and other gene-related features and gives a visual display of explanatory notes on the bayberry genome (Fig. 3). Users can read the gene model on scaffolds from the genome browser. For example, if the user selects the genomic region of 4,130,542 bp to 4,411,741 bp on Scaffolds.01 for browsing, all of the genes in this zone will display as requested (Fig. 3A). If the user clicks on ‘GENE_019554’, the next level appears with specific gene data, such as the CDS, exons, mRNAs, and other features (Fig. 3B).

#### Germplasm resource data

**Fig. 4:**
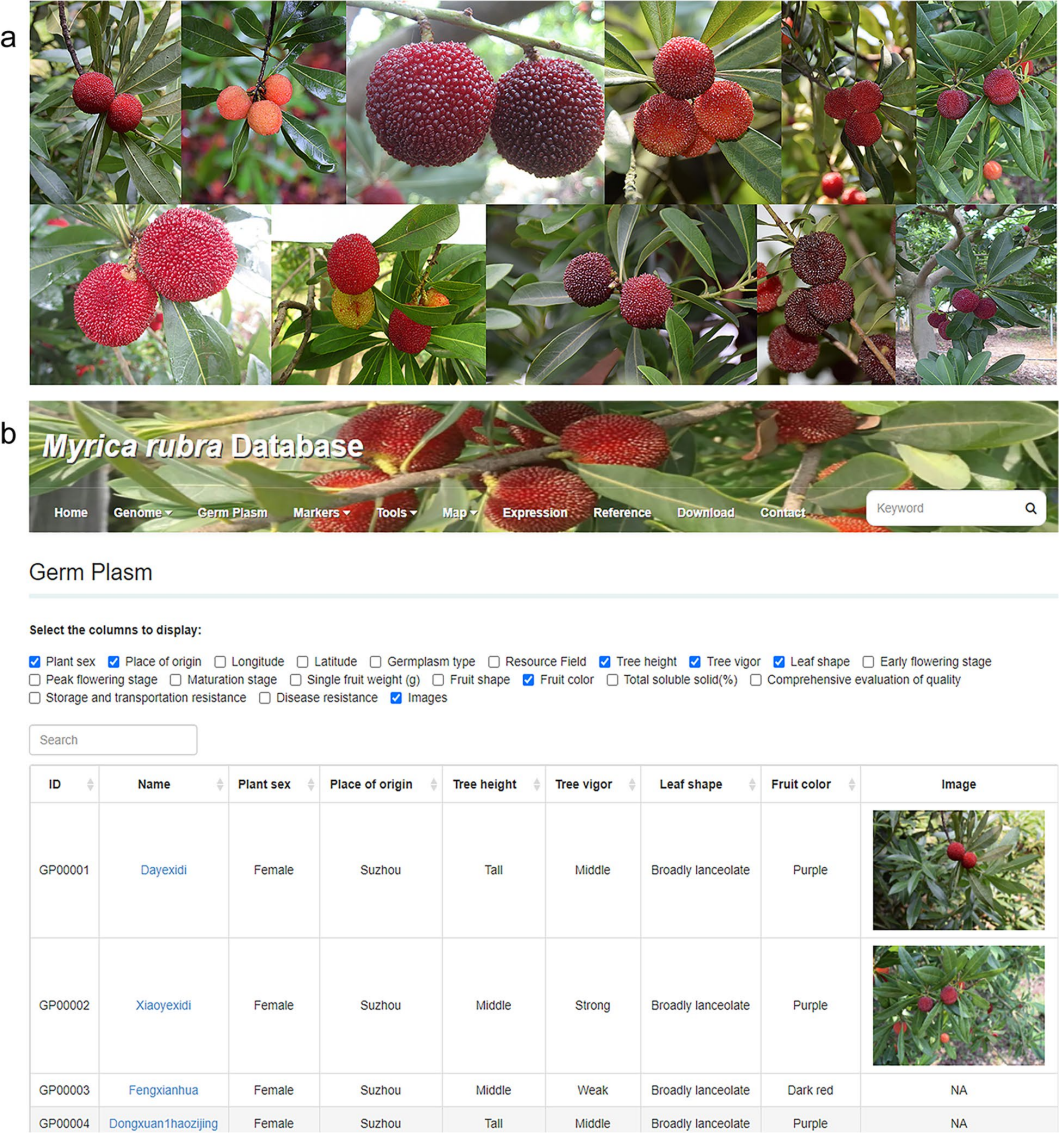
Brief view of the ‘germplasm’ module. **A** Images of fruits with various shapes, colors and qualities in the bayberry germplasm pool. **B** A snapshot of the phenome database in the Bayberry Database

At present, the germplasm module of the Bayberry Database contains phenotypic data of bayberry along with images of the fruits. The bayberry fruit shapes changed little, but there were differences in tree vigor, leaf type, fruit color, and single fruit weight, and the stages of early bloom and full bloom were slightly variable among the different cultivars. Most of the fruit shape types of bayberry appear in the germplasm module, which can be browsed and downloaded by users (Fig. 4A). The image data mainly exhibit distinctions in the shape, size and color of bayberry fruit. For example, the fruit color of GP00101 ‘Zaojia’ is dark purple, and the fruit shape is intimate circle; the fruit color of GP00064 ‘Dongkui’ is red, and the fruit shape is round. The module also provides a series of information on approximately 102 bayberry cultivars, which includes information such as the name, plant sex, place of origin, and level of disease resistance (Fig. 4B). Users can enter the ID or name of cultivars in the search bar to browse and fetch information regarding a variety of bayberry cultivars.

#### Marker data

In the molecular marker module, 59 pairs of gSSR molecular markers were collected. Based on the genomic sequence information of bayberry [8], in total, 43,842 SSR loci have been identified, and 59 pairs of gSSRs have been cluster analyzed; the polymorphism information content (PIC), Nei’s gene diversity (h*), effective number of alleles (Ne), and number of alleles (Na) can be found in the database [21].

#### Tool search

**Fig. 5:**
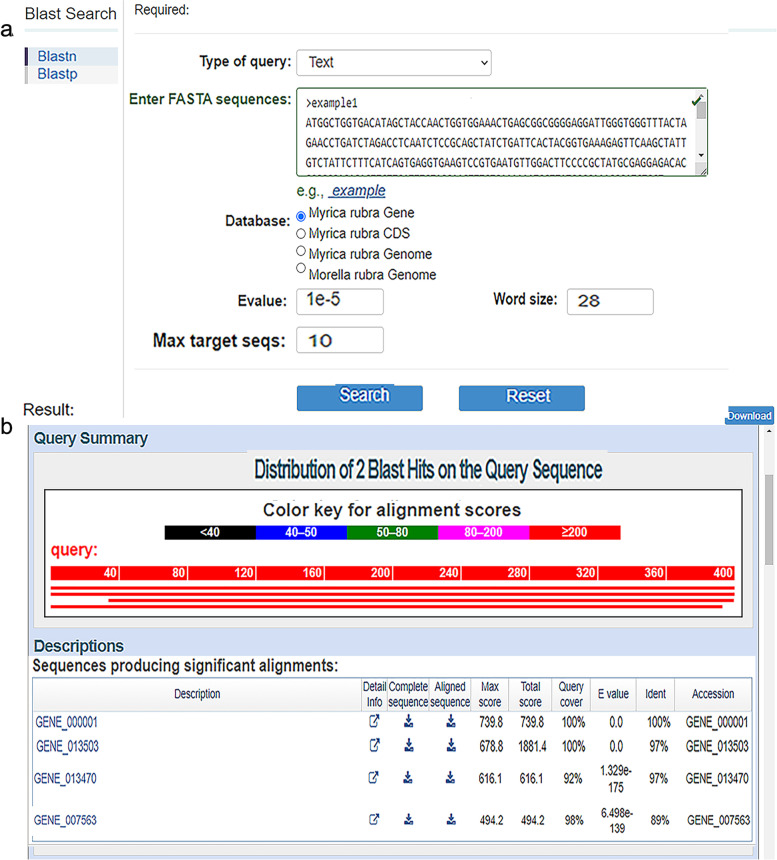
Screenshot of ‘Blast Search’ in the tools module. **A** Demonstration of the ‘Blast Search’ dialog box. **B** Query results after inputting the FASTA sequence and selecting the database type

**Fig. 6:**
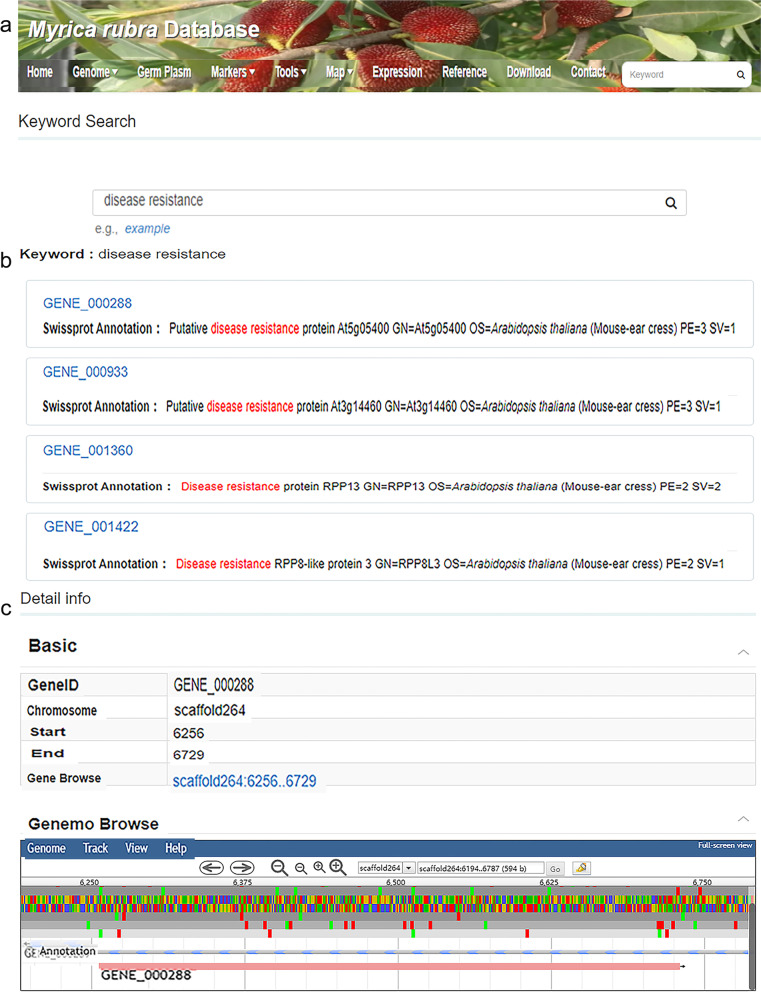
Screenshot of ‘Keyword Search’ in the tools module. **A** Enter a keyword in the dialog box, such as ‘disease resistance’. **B** According to the keyword information, the database screened out the related genes. **C** The interface after clicking on ‘GENE_000288’

The tools module supplies users with diverse methods for genome searching. When the user clicks on ‘Tools’, the column title tags ‘Enrichment Analysis’, ‘Sequence Fetch’, ‘Keyword Search’, and ‘Blast Search’ are displayed. When the user clicks on ‘Blast search’, an extra layer appears with two options, ‘Blastp’ and ‘Blastn’. Protein sequences can be compared with sequences in the protein database to identify the sequence from a distant source with Blastp, and given nucleic acid sequences can be compared with sequences in the nucleic acid database with Blastn. Users can enter a FASTA sequence in the box, select the database type (Fig. 5A), and click on the ‘Search’ button to obtain the nucleotide sequence comparison results; the sequence comparison results of Blastn or Blastp can be downloaded from the database (Fig. 5B). ‘Keyword Search’ is a more practical function in the tools module (Fig. 6). By inputting the desired keywords, the user can find genes with that keyword content in the database. For example, if the user enters ‘disease resistance’ in the box and clicks on the ‘Search’ button (Fig. 6A), then the genes related to disease resistance will be displayed, such as ‘GENE_000288’ and ‘GENE_000325’. (Fig. 6B). Detailed information on the genes can be obtained after clicking on their name (Fig. 6C). In the ‘Sequence Extraction’ function, users can input the starting fragment of the scaffold to obtain the DNA sequence corresponding to the genomic coordinates. Gene functional enrichment analysis is a method to identify types of genes or proteins that are overrepresented in a large group of genes or proteins and may be related to disease phenotypes. The method employs statistical approaches to identify significantly enriched or depleted groups of genes. Transcriptomic technologies and proteomics results are often used to identify thousands of genes that are used for the analysis. There are two enrichment analysis methods in this database: ‘GO’ and ‘KEGG’. Users can select the corresponding analysis methods according to their own needs.

#### Genome map

The map module contains two kinds of bayberry gene maps. One is the red bayberry genetic haploblock (HB) map, which was constructed using RAD tag sequencing technology. The HB markers for the l m × l l segregation type are shown in red, those for nn × np are shown in green, and the ab × cd segregation type is shown in blue. The HBs in the centromere are indicated in bold. The other map is the alignment of female (a) and male (b) bayberry containing assembled scaffolds with the SNP marker linkage genetic map [22]. The map module contains the evolutionary genetic map of bayberry, a total of 4270 gene families were shared by these four species, and 891 gene families were unique to bayberry. Based on 560 single-copy protein sequences, a phylogenetic tree was constructed for nine species (*M. rubra*, *Prunus mume*, *Citrullus lanatus*, *Morus notabilis*, *Fragaria* × *ananassa*, *Citrus sinensis*, *Carica papaya*, *Arabidopsis thaliana*, and *Solanum lycopersicum*) by using PhyML 4.0 [23]. Three species, *M. rubra*, *P. mume*, and *M. notabilis,* were clustered in a subclade that was most likely derived from a common ancestor approximately 93.95 Mya, whereas *P. mume* and *M. notabilis* diverged 82.41 Mya, and *C. lanatus* diverged from an ancestor shared with *M. notabilis, P. mume*, and *M. rubra* approximately 97.67 Mya [8]. In addition, comparative genomic analysis of bayberry and other fruits was also performed in the map module. A total of 13,216 unique gene families were produced by gene similarity clustering of the 26,325 predicted genes for *M. rubra* with those of *M. notabilis, F. × ananassa, Vitis vinifera*, and *S. lycopersicum*. We found that the five species shared 4074 gene families, and 712 gene families were unique to *M. rubra*. A total of 12,266 unique gene families were yielded by gene similarity clustering of the 26,325 predicted genes for *M. rubra* with those of *Oryza sativa*, *A. thaliana*, and *Cicer arietinum* [8].

#### Gene expression data

The gene expression module of the Bayberry Database contains transcriptome data of the fruit development process of two Chinese bayberry cultivars ‘Zaojia ‘and ‘Dingao Bayberry’ post pollination 10, 20, 30, 40, 50 days. Users can click on the sample name to obtain detailed information regarding the gene, such as gene expression, gene function, and cDNA sequence. The database offers a platform for visualization and download of specific gene data of bayberry, which can assist researchers and breeders in performing in-depth experiments.

#### Reference data

The reference module of Bayberry Database currently contains 126 publications supporting the aforementioned information from 1985 to 2020, which can be browsed and downloaded freely with the authors’ permission. Among these publications, the completion and publication of bayberry genome sequencing results is helpful to understand the structure and function of the bayberry genome and provide an important reference for exploring the origin and evolution of species, the development and application of molecular markers, and the location and cloning of functional genes [8]. The process of molecular breeding may be accelerated by genome data. Based on the genomic sequence information of bayberry [8, 9], the 43,842 SSR loci in 623 scaffold sites were identified [21]. The genetic relationship between main cultivars and male plants of bayberry were analyzed by RAPD and ISSR markers, which provides theoretical guidance for the selection of male parents in bayberry cross breeding [24].

The Bayberry Database was made available online in June 2019 and is currently in the initial version. With the increasing number of innovative data in the Bayberry Database, various types of molecular markers (e.g., SSRs, indels, and QTLs), genome resequencing and new publications, the database will be continuously optimized with time. The data classification, integration and update will be emphasized in the Bayberry Database, and a large quantity of comprehensive data will be conveniently provided in the near future.

Some new resequenced genome, transcriptome and molecular marker data will be added to improve the functions of some modules of the Bayberry Database. The Synteny Viewer module will be added to the Bayberry Database because there is no publicly available database of the bayberry genome. This module has been used in the Genome Database for Rosaceae (https://www.rosaceae.org) [25] and the Citrus Genome Database (https://www.citrusgenomedb.org) [26]. The primary function of the module is to view genome synteny and homologous gene pairs between different species, and the gene synteny clusters between two genomes can be dynamically presented with Genome Synteny Viewer as a circular plot. The new map sets, phenome data, and novel search and download options will also be added. The long-term goals of the Bayberry Database include constructing an integrated, intelligent and comprehensive information system that integrates more genomic, transcriptomic and metabolomic data. With more bayberry cultivars available in omics data, the database will benefit both researchers and fruit-tree breeders.

## Conclusions

The web address of the database is as follows http://www.bayberrybase.cn/. The Bayberry Database contains nine central functional domains: home, genome, germplasm, markers, tools, map, expression, reference, and contact. The *Myrica rubra* database is an intelligent, interactive, and user-friendly system that enables researchers, breeders and horticulturalists to browse, search and retrieve relevant and useful information and facilitates genomic research and breeding efforts concerning *Myrica rubra*. This database will be of great help to bayberry research and breeding in the future.

## Methods

The bayberry database was deployed in Ubuntu 16.04 operation system and developed by AKKA 2.12 (web server), MySQL 5.7.26 (database server), Scala 2.12.2 and SBT 0.13.18. All data in database were managed and stored by MySQL Database Management System. The query function was enforced based on Slick 3.3.0 middleware tier. To visualize the genome, we used the Jbrowser 1.16.6. The website interface components were designed and implemented by the Bootstrap 3.3.0 and Play Framework 2.6.25. The website has been tested in several popular web browsers, including Firefox, Google Chrome and Internet Explorer. Source code is available in github https://github.com/Westsyan/Myrica_rybra_database.

## Data Availability

Source code is available in github https://github.com/Westsyan/Myrica_rybra_database. The database and web interface can be accessed at http://www.bayberrybase.cn/.
